# Genetic differentiation and population structure of *Anopheles funestus* from Uganda and the southern African countries of Malawi, Mozambique, Zambia and Zimbabwe

**DOI:** 10.1186/s13071-020-3962-1

**Published:** 2020-02-18

**Authors:** Martha A. Kaddumukasa, Jane Wright, Mbanga Muleba, Jenny C. Stevenson, Douglas E. Norris, Maureen Coetzee

**Affiliations:** 1 Wits Research Institute for Malaria, School of Pathology, Faculty of Health Sciences, University of the Witwatersrand, Johannesburg, South Africa; 2 Inqaba Biotechnical Industries, PO Box 14356, Hatfield, 0028 Pretoria, South Africa; 3 Macha Research Trust, Choma District, Zambia; 4 Southern and Central Africa International Centers of Excellence in Malaria Research, Department of Molecular Microbiology and Immunology, John Hopkins Malaria Research Institute, Johns Hopkins Bloomberg School of Public Health, Baltimore, USA; 5 Center for Emerging Zoonotic and Parasitic Diseases, National Institute for Communicable Diseases, Johannesburg, South Africa

**Keywords:** *Anopheles funestus*, Population differentiation, Malaria, Microsatellites, East Africa, Southern Africa

## Abstract

**Background:**

*Anopheles funestus* (*s.s.*) is a primary vector of the malaria parasite *Plasmodium falciparum* in Africa, a human pathogen that causes almost half a million deaths each year. The population structure of *An. funestus* was examined in samples from Uganda and the southern African countries of Malawi, Mozambique, Zambia and Zimbabwe.

**Methods:**

Twelve microsatellites were used to estimate the genetic diversity and differentiation of *An. funestus* from 13 representative locations across five countries. These were comprised of four sites from Uganda, three from Malawi and two each from Mozambique, Zambia and Zimbabwe.

**Results:**

All loci were highly polymorphic across the populations with high allelic richness and heterozygosity. A high genetic diversity was observed with 2–19 alleles per locus and an average number of seven alleles. Overall, expected heterozygosity (H_e_) ranged from 0.65 to 0.79. When samples were pooled three of the 12 microsatellite loci showed Hardy–Weinberg equilibrium. Unsupervised Bayesian clustering analysis of microsatellite data revealed two clusters with *An. funestus* samples from Mozambique, Uganda and Zambia falling into one group and Malawi and Zimbabwe into another. The overall genetic differentiation between the populations was moderate (F_ST_ = 0.116). Pairwise differentiation between the pairs was low but significant. A weak but significant correlation was established between genetic and geographical distance for most populations.

**Conclusions:**

High genetic diversity revealed by the loci with low to moderate differentiation, identified two clusters among the *An. funestus* populations. Further research on the population dynamics of *An. funestus* in east and southern Africa is essential to understand the implications of this structuring and what effect it may have on the efficient implementation of mosquito vector control strategies.
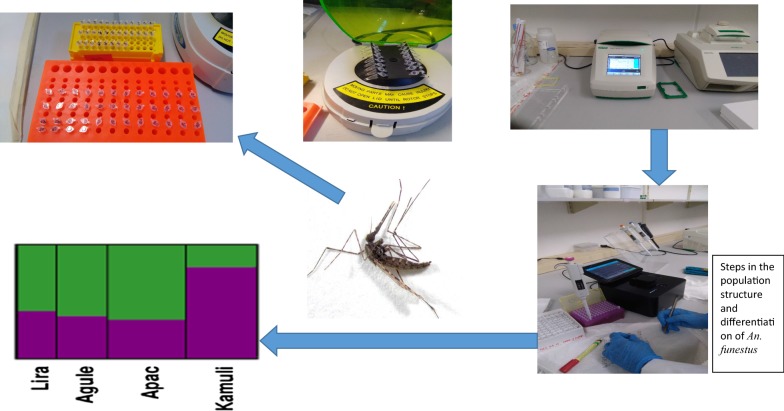

## Background

*Anopheles funestus* (*sensu stricto*) is present throughout most of sub-Saharan Africa and is one of the four most important vectors of human malaria parasites [1–3]. In parts of east and southern Africa, its importance as a vector exceeds that of members of the *An. gambiae* complex [1, 4]. The traditional methods of vector control, insecticide-treated bednets and indoor residual house spraying, have been very effective in controlling *An. funestus* due to its strong association with humans and human habitations [1, 2]. However, the development of insecticide resistance across the region [5, 6] has seen vector control programs compromised in some places [7–9]. This has also played an important role in the slow-down of the gains made in malaria control over the past 10 years in Africa [10, 11]. To achieve the goals of malaria elimination and eradication, not only are new products and interventions needed, but monitoring the effectiveness of all vector control interventions is crucial.

Mosquito vector surveillance covers the monitoring of all aspects of mosquito biology, from geographical distribution to the more complex genetic diversity within and between populations. Studies of population genetics provide insight into gene flow between mosquito populations and through that, the likelihood of the spread of genes (e.g. those conferring insecticide resistance) across geographical regions. In east and southern Africa, studies focused on the population structure of *An. funestus* have been carried out in Uganda [12–14], Malawi [12, 15–17], Kenya [18], Tanzania [19], Mozambique [12, 16, 17] and Zambia [16]. These and other studies have revealed various levels of differentiation over the African continent, from no or very low structure to high population structure of *An. funestus* [12–19].

Understanding the gene flow dynamics of populations of malaria vector mosquitoes is important for strategic planning of vector control interventions. In this study, the population structure of *An. funestus* was examined using specimens from one country in east Africa (Uganda) and four countries in southern Africa (Malawi, Mozambique, Zambia and Zimbabwe). Twelve microsatellite markers distributed across all five chromosome arms were used to address the questions: (i) Is there any evidence for population genetic subdivision within this collection of *An. funestus*? and (ii) What is the maximum amount of differentiation observed within each selected site? Population structure analysis quantifies the amount of genetic exchange that occurs between sample sets and provides insight on how these underlying genetic components may be used to address biological phenomena such as insecticide resistance, parasite transmission and dispersal of the mosquito.

## Methods

### Mosquito collection study sites

**Fig. 1:**
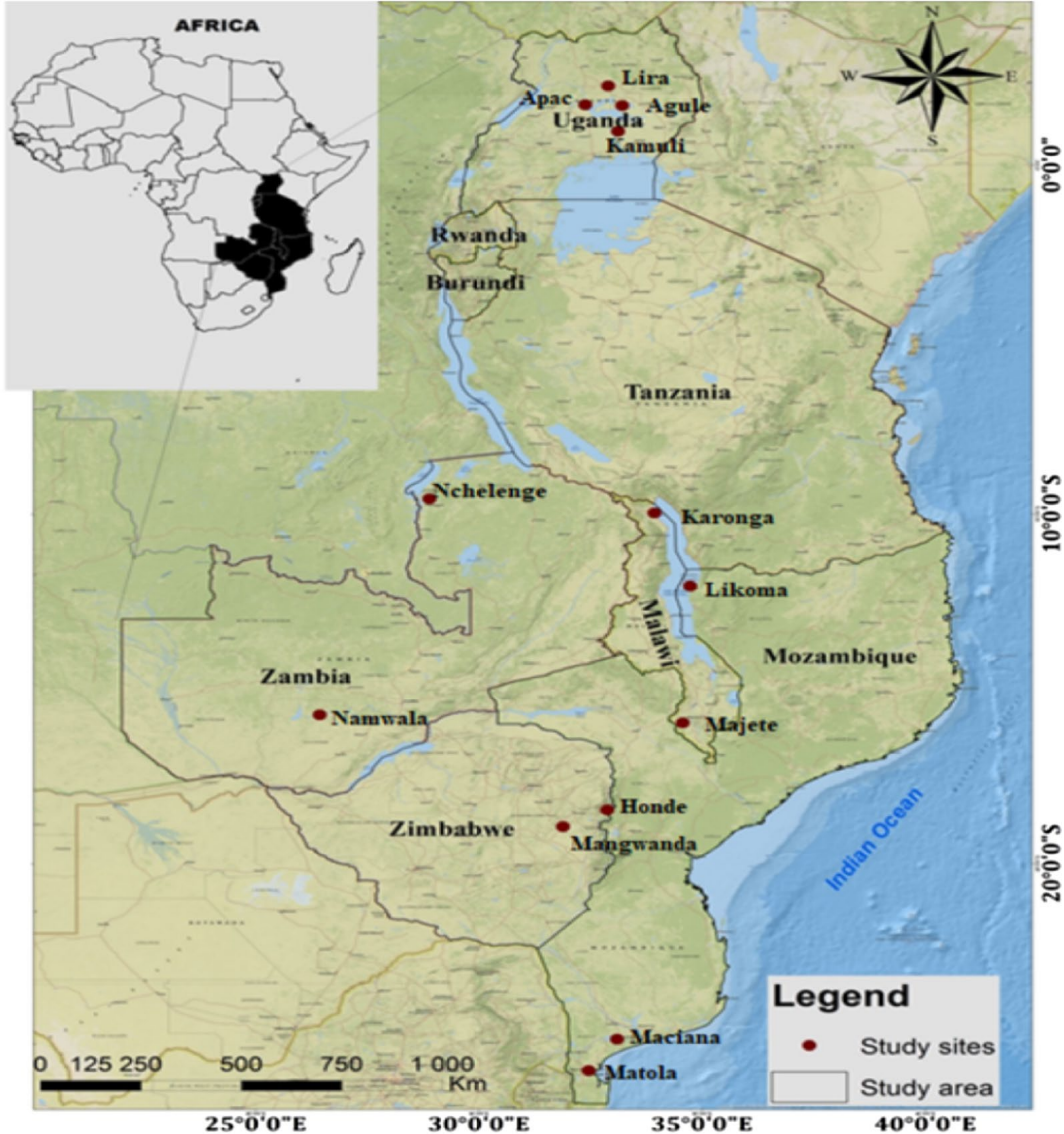
Mosquito collection sites from five African countries

Preserved mosquito samples from selected sites (Table 1) in five African countries (Malawi, Mozambique, Uganda, Zambia and Zimbabwe) (Fig. 1) were used. From Malawi, sample collections were from three sites; Karonga, Majete and Likoma Island. Karonga is found in the far north of Malawi, Majete is an area near Majete Wildlife Reserve in the south-west of Malawi, and Likoma is an island in the north eastern corner of Lake Malawi close to Mozambique. In Malawi, Karonga has two seasons, a dry season from May to mid-November and a wet season from mid-November to April, temperature varies between 16.11–33.3 °C and humidity varies between 54–79%. In Likoma, the wet season starts from November to April and the rest of the year from May to October is almost dry. Temperatures vary between 22.4–31.4 °C and humidity varies between 43.7–83.6%. In Majete there are two distinct seasons, the dry season from May to September and the wet season from November to April. Temperature varies between 22–34 °C and the average humidity is 71%.

**Table 1:** Country and sampling sites, number of specimens collected, collection method, and malaria transmission intensity

Country	Site	Coordinates^a^	*n*	Clade I	Clade II	Method^b^	Collection date	Transmission intensity
Malawi	Karonga	− 9.933, 33.933	13	13	–	IAC	December 2007, 2010	High (> 1 case per 1000 population)
	Majete	− 15.785, 34.008	26	26	–	IAC	May 2012	High (> 1 case per 1000 population)
	Likoma	− 12.067, 34.733	21	21	–	IAC	May 2010	High (> 1 case per 1000 population)
Mozambique	Maciana	− 25.449, 32.781	34	28	6	IAC	April 2011	High (> 1 case per 1000 population)
	Matola	− 25.962, 32.459	36	30	6	IAC	October 2012	High (> 1 case per 1000 population)
Uganda	Agule	1.667, 33.817	13	13	–	HLC	April 2013	High (> 1 case per 1000 population)
	Apac	1.983, 32.533	20	20	–	IAC	August 2016	High (> 1 case per 1000 population)
	Lira	2.235, 32.909	10	10	–	HLC	April 2013	High (> 1 case per 1000 population)
	Kamuli	0.947, 33.120	20	19	1	IAC	August 2016	High (> 1 case per 1000 population)
Zambia	Nchelenge	− 9.345, 28.734	30	22	8	IAC	April 2013, 2015	High (> 1 case per 1000 population)
	Namwala	− 15.750, 26.450	30	18	12	IAC	January 2012	Low (< 1 case per 1000 population)
Zimbabwe	Honde	− 18.497, 32.853	35	35	–	IAC	February 2013	Seasonal and geographic variation in malaria transmission
	Mangwanda	− 18.570, 31.526	35	35	–	IAC	March 2013, May 2014	Seasonal and geographic variation in malaria transmission

^a^Latitude, longitude

^b^Human landing and indoor aspiration collections were done in the living/sleeping areas of the huts IAC, Indoor aspiration catch, HLC, Human Landing Catch

*Note*: Reference https://www.who.int/malaria/publications/country-profiles/profile_

*Abbreviation*: n, number of specimens collected

In Mozambique, samples were collected from Maciana and Matola, near Maputo in southern Mozambique. Matola is the largest suburb of Maputo and lies on the coastal plain north of Espirito Santo estuary. In Matola, found 42 m above sea level, the climate is characterized by a warm and wet season from December to March, and a dry and cold season from June to August. Precipitation averages 1050 mm (41 inches) per year, with abundant rainfall from December to March, and rare rains from May to October. Temperature varies between 15–34 °C and relative humidity ranges between 28–89%. Maciana, at 68 m above sea level is characterized by a warm and wet season from November to April, and a dry and cold season from May to October. Precipitation averages 815 mm (32 inches) per year, most of which falls from November to March. The rainiest month is January (170 mm). Temperature varies between 11–34 °C and relative humidity ranges between 59–67%.

Uganda experiences a warm tropical climate with heavy rains in March–May and September–November for the South. In northern Uganda, the wet season extends from April to November with peaks in April and August. Samples were obtained from Agule, Apac, Lira and Kamuli districts in North and central Uganda. Agule and Apac were originally one district so do not have much variation in climate characteristics. Agule lies 1034 m above sea level. Temperature varies between 20–34 °C and relative humidity varies between 28–88%. Apac lies on 1040 m above sea level with a tropical climate, over the course of the year, the temperature varies between 18–35 °C. Temperatures are highest in February, with an average of around 25.1 °C, whilst July is the coldest month of the year with an average of 22.1 °C. Precipitation over this area, averages 1305 mm. Humidity varies between 21% and 69%. Lira lies at 1091 m above sea level and has an average temperature of 23.2 °C and a yearly average precipitation of 1376 mm. The wet season runs from April to mid-November and with two dry seasons (December to February and June to August). February is the warmest month of the year with an average temperature of 33 °C. July has the lowest average temperature, with an average of 21.6 °C. Overall, the average humidity for Lira is 58%. On the other hand, Kamuli lies 1185 m above sea level and temperature varies between 15.5–32 °C. March is the warmest month of the year, with an average temperature of 22.7 °C, whilst July has the lowest average temperature of 20.7 °C. There is a great deal of rainfall in Kamuli, even in the driest month (January) with 63 mm of rainfall. April is the wettest with an average of 193 mm. Average rainfall is 1325 mm over the year. Humidity varies between 62–95%.

Zambian collections were from Nchelenge and Namwala. Namwala is located in the north-western corner of the southern province next to Kafue National Park. Zambia has a tropical climate, which experiences wet and dry periods. In Namwala, the wet season lasts from mid-November to March and the dry season from end of November to April. The cool season is from May to August with temperatures varying between 10–27 °C and the dry season from September to November with temperatures around 26 °C to 37 °C and humidity between 19–75%. Some days are particularly dry where humidity can be as low as 0%. Nchelenge district is located near the Democratic Republic of Congo next to Lake Mweru. For Nchelenge, the wet season lasts from September to May and the dry season from end of May to September. The cool season is from December to April with temperatures varying between 15–27 °C. The hot season is from September to October with temperatures around 22 °C to 34 °C and humidity varying between 22–89%. In Nchelenge, humidity can also reach a low of 0%.

In Zimbabwe, samples were obtained from Honde and Mangwanda. Both sites are located within Mutasa district. In Zimbabwe, Honde, from late October to around the end of April, the weather is hot and humid. Temperatures rise up to 28 °C and is when most of the convectional rainfall is received. Average rainfall is 1150 mm. From May to the beginning of July, the temperatures are very low, up to 2 °C, whilst August is very windy. During September to October very hot temperatures are recorded, where the maximum temperature may average 30 °C. Humidity varies between 11–45%. In Mangwanda, the climate is warm and temperate. The summers have much more rainfall with an average of 809 mm. From May to August the temperatures are very low, varying between 9–16 °C, while August is very windy. From September to December, it is very hot and the temperatures range between 16–28 °C. The average humidity is 73%.

### Mosquito collection and identification

Specimens were collected using indoor aspirations for all mosquitoes mainly in the living/sleeping area of the houses (huts), but for two sites human landing catches were carried out (Table 1). Anopheline mosquitoes were identified morphologically [2] and preserved individually in 0.6 ml microcentrifuge tubes with silica gel and stored at room temperature until processing. Molecular identification was carried out using the standard method of Koekemoer et al. [20] and the clade TaqMan assay of Choi et al. [21].

### DNA extraction and microsatellite genotyping

Genomic DNA extractions were performed on the legs of the individual *An. funestus* mosquitoes using a DNA extraction kit prepGEM^®^Insect (Cat. No. PIN0050; ZyGEM, Hamilton, New Zealand) following the manufacturer’s instructions. In short, a leg of an individual mosquito was homogenized with reagents, incubated for 15 min at 75 °C and at 95 °C for 5 min. A 10 µl elution of genomic DNA was obtained for each sample from the extraction.

Twelve microsatellite loci were selected from previously published *An. funestus* sequence data [22–25], based on their high polymorphism and no evidence for null alleles. These microsatellites were: FUNF and AFUB12 on chromosomal arm 3L; AFND19, AFND20 and AFND41 on 3R; AFUB10, AFUB11, FUNL and AFND23 located on 2L; AFUB3 and FUNO located on 2R; and FUNQ on X (Table 2). PCR amplification of the microsatellite loci was carried out individually in a 25 µl reaction volume, using 5–10 ng of template DNA. The reaction mixture contained 2.5 µl (1× PCR) buffer (Takara Bio Inc, Shiga, Japan), 1.5 mM MgCl_2_ (Takara Bio Inc), 0.2 mM of each dNTP (Takara Bio Inc), 10 pmol of each primer, 0.1 U of Taq DNA polymerase (Takara Bio Inc). The forward primer was labelled at the 5′-end with either PET, HEX or 6-FAM fluorescent markers to allow multiplex electrophoresis. Amplification was carried out with a T100™ thermal cycler (Bio-Rad, Hercules, USA), under the following conditions: an initial denaturation step at 94 °C for 2 min; 36 cycles of 30 s at 94 °C, 30 s at 54 °C, 30 sec at 72 °C; and a final elongation step of 10 min at 72 °C. Fragment analysis was performed commercially at Macrogen (Seoul, South Korea) with an ABI PRISM 377 (Thermo Fisher Scientific, MA, USA). Alleles were sized relative to an internal size standard using Peak Scanner v1.0 (Applied Biosystems, Toulouse, France). Output files (*.fsa) generated by the autosequencer were loaded into Peak Scanner™ software. Determination of fragment size was performed using default settings, with GS500LIZ specified as size standard and ‘PP - Primers Present’ in the ‘Analysis Method’ column. Peak information showing height, area and size were labelled and scored. The resulting fragment sizes were used for further analyses.

**Table 2:** Primer sequences and characteristics of 12 polymorphic microsatellite loci

Locus	Chromosome arm	Repeat motif	No. of alleles	Allele size	Primer sequence (5′-3′)	Reference
FunL F	2L	(GT)_8_	12	181–197	HEX-AACAGTGGAAGGCAAATTGC	Cohuet et al. [22]
FunL R					GCACGGTTACCACTGCTCA	
AFUB10 F	2L	(GCT)_3+2+4+5_	6	195–210	PET-TGTCCATGTACAACCGCAAC	Sharakov et al. [23]
AFUB10 R					TTCTCCAGCATCATCAGCAC	
AFUB11 F	2L	(CTG)_3+5+2+2_	4	188–191	PET-CAGTTTCTGCGTGGAGGAAT	Sharakov et al. [23]
AFUB11 R					AGCAGCTGATGAGCCATCTC	
AFND23 F	2L	(GT)_11_	11	133–157	6-FAM-TTTGATCGACGGACTAGTGTGT	Schemerhorn et al. [25]
AFND23 R					GGTTTGATGGGTGGAAAC	
FunO F	2R	(CA)_6_T(AC)_4_	10	110–132	PETGCACACATTTCAGGCAGC	Cohuet et al. [22]
FunO R					GCCCACATTCTGCACCTT	
AFUB3 F	2R	(CAG)_2+3+2_	5	171–195	VIC- GGGAAGGATTCGACCTTAGC	Sharakov et al. [23]
AFUB3 R					GCCGCCATTTAGTAGCAGTT	
FunF F	3L	(TG)_9_	7	104–118	6-FAM –GCCTTCAGTTTCGATTGGCG	Cohuet et al. [22]
FunF R					AATAAGATGCGACCGTGGC	
AFUB12 F	3L	(AGG)_7_(TG)_4_	3	152–158	VIC –TGGGGAACTGGTCGTTAGAG	Sharakov et al. [23]
AFUB12 R					CTGGTGATGGGATTGAGGAT	
AFND19 F	3R	(AG)_12_(TG)_5_	8	251–285	HEX –GCAAGCTGTACGCAGAGAG	Sharakov et al. [24]
AFND19 R					ATCGATGGGAGTTATTATACGC	
AFND20 F	3R	(GAG)_4+2+2+2_(TGG)_3_	2	239–242	VIC- CGGCGCAGGTTTAGTAGC	Sharakov et al. [24]
AFND20 R					CCCTCGCTTTCCTCATAAAA	
AFND41 F	3R	(CA)_3_TC(CA)_6_	6	222–246	6-FAM AGAACATATGGCAAATCGAC	Schemerhorn et al. [25]
AFND41 R					GAAAGACTTGTCGGACGTG	
FunQ F	X	(TG)_9_	7	84–98	HEX –GCAAACTGCTAGTAAATGTTTCC	Cohuet et al. [22]
FunQ R					ACATTTCCACAATTTGCGC	

### Microsatellite diversity, differentiation and structure analysis

Microsatellite allele and genotype frequencies were determined using Arlequin 3.5 [26] and FSTAT 2.9.3 [27]. These frequencies were then used to assess deviation from Hardy–Weinberg equilibrium at each locus, each population sample, and overall as indicated by the inbreeding coefficient (F_IS_). Linkage disequilibrium between pairs of microsatellite loci was assessed using FSTAT 2.9.3 [27]. The level of genetic differentiation between sampling groups was assessed using F_ST_ [28]. Pairwise F_ST_ between sampling groups and their significance was assessed using the randomization approach implemented in FSTAT 2.9.3 with Bonferroni-adjusted *P*-values.

The significance of genetic differentiation between populations based on allelic distribution across populations was examined using a Fisher exact test with FSTAT 2.9.3 [27]. The pairwise F_ST_ was assessed by estimating Wright’s F-statistics, calculated according to Weir & Cockerham [28]. A locus by locus analysis of molecular variance (AMOVA) [29] was performed using Arlequin 3.5 to determine the relative contribution of within-sampling groups and between sampling group’s genetic diversity to the overall genetic diversity. Finally, population structure was inferred using a Bayesian model-based clustering algorithm to assign individuals (probabilistically) to clusters without prior knowledge of population units and limits in STRUCTURE 2.3.4 [29, 30]. This model calculates the probabilities of each individual for each subgroup within which Hardy–Weinberg (H–W) equilibrium and linkage equilibrium are minimized. Using the STRUCTURE program, the numbers of distinct genetic clusters in the data set (K) are estimated from 1 to 10 by posterior log probability of data under K, Ln [Pr (X|K)]. Each run was carried out with 1,000,000 iterations after a ‘burn-in’ period of 100,000, using the admixture model and correlated allele frequencies. To check for convergence of the Markov Chain Monte Carlo (MCMC), 5 replicates for each value of K were checked for the consistency of results [29, 30]. Based on allele diversity, individuals with unique alleles were grouped together into assumed populations (K) which is pre-determined. The K value with the maximum posterior probability Pr (X|K) value was retained and assumed to be the most probable number of clusters in that population. The estimated number of clusters (K) is taken to be the value of K with the highest Pr (X|K). All obtained files were run through Structure Harvester [31] followed by CLUMPP [32] and DISTRUCT [33]. The visual representations of the clusters were viewed in Ghostview, a graphical interface for Ghostscript (https://www.ghostscript.com). As an estimate of gene flow, the number of migrants per population per generation (Nm ≈ (1 − F_ST_)/4F_ST_ was conducted [34]. The correlation between genetic and geographical distances was assessed by the regression of F_ST_/(1 − F_ST_) on the logarithm (ln) of geographical distance, in GenALEx 6.503 [35, 36].

### Principal coordinates analysis

For the majority of the time, principal coordinates analysis (PCoA) uses Euclidean distance between two points that are being compared. To visualize the genetic relatedness among individuals, we performed PCoA after all the preliminary analyses using the GenALEx 6.503 [35, 36] program to explore the relationships between the *An. funestus* samples. PCoA was conducted based on mosquito genotypes. The genotypes at each site were transformed with natural log (ln (x + 1)). Then, the sites on the PCoA ordination map were marked as groups from the cluster analysis extracted from the collected sites. PCoA is an indirect gradient analysis method for seeking the strongest linear correlation structure among variables [35, 36] and it is a technique widely used for reducing the dimensions of multivariate problems. In the PCoA, eigenvalues, which explain a portion of the original total variance, were calculated. Each axis score using the eigenvector, which contains the coefficients of the linear equation for a given axis, was shown in an ordination [35, 36].

## Results

### Mosquito identification

A total of 323 *An. funestus* individuals were identified from the 13 localities. Both Clades I and II were found (Table 1), with Clade I found in all sites but Clade II only in Mozambique and Zambia. A repeat of the single Clade II specimen from Kamuli in Uganda was performed and it was still assigned to Clade II. However, many more *An. funestus* samples need to be examined, given that the TaqMan assay has been shown to have some limitations in its accuracy [37].

### Genetic diversity

Levels of microsatellite polymorphism were moderate to high for all the twelve loci. One site from Malawi and two from Uganda had small sample sizes (less than 15 specimens, Table 1) but this did not affect the level of microsatellite polymorphism (*P* > 0.05) compared with previous studies. Overall, the mean observed heterozygosity (H_o_) values ranged from 0.26 to 0.42 and expected heterozygosity (H_e_) from 0.65 to 0.79 (Additional file 1: Table S1). These heterozygosity values were not significantly different among populations (*P *= 0.36). A summary of the mean variation of microsatellite loci from each country is presented in Table 3. Overall allelic richness averaged seven alleles per locus with a range of 2–19 (Table 2). Locus FUNQ had the lowest number of alleles (*n* = 2) and AFUB11 the greatest number of alleles (*n* = 19).

**Table 3:** Allelic richness, heterozygosity, fixation and inbreeding indices for 12 microsatellite loci in populations of *An. funestus* collected from Malawi, Mozambique, Zambia, Zimbabwe and Uganda

Index	Na	Ho	He	F_ST_	F_IS_	Na	Ho	He	F_ST_	F_IS_	Na	Ho	He	F_ST_	F_IS_	Na	Ho	He	F_ST_	F_IS_
Locus	FUNQ (X)					FUNL (2L)					AFUB10 (2L)					AFUB11 (2L)				
Malawi (*n* = 60)	4.67	0.12	0.76	0.20	1.00	7	0.37	0.82	0.13	0.44	8.67	0.27	0.84	− 0.02	0.68	6.00	0.00	0.80	− 0.06	1.00
Mozambique (*n* = 70)	3.00	0.34	0.56	0.06	0.33	7.5	0.61	0.82	0.02	0.18	4.00	0.60	0.75	0.01	− 0.21	na	na	na	na	na
Uganda (*n* = 63)	3.25	0.00	0.63	0.10	1.00	6.25	0.60	0.76	0.13	− 0.22	5.75	0.05	0.72	− 0.02	0.82	4.25	0.13	0.62	0.02	0.69
Zambia (*n* = 60)	3.00	0.17	0.61	0.00	0.81	12	0.58	0.86	− 0.02	0.21	na	na	na	na	na	16.50	0.48	0.89	0.04	0.39
Zimbabwe (*n* = 70)	5.00	0.06	0.73	0.21	0.93	4	0.23	0.67	− 0.02	0.39	6.00	0.15	0.73	0.01	0.67	13.00	0.38	0.86	0.02	0.55

Locus	FUNF (2R)					FUNO (2R)					AFND23 (2R)					AFUB3 (2R)				
Malawi (*n* = 60)	4.67	0.49	0.70	− 0.02	− 0.05	5.33	0.47	0.73	0.04	0.11	3.67	0.27	0.68	0.05	0.16	5.33	0.95	0.71	− 0.02	− 0.64
Mozambique (*n* = 70)	3.00	0.23	0.65	0.16	0.43	5.50	0.39	0.71	0.25	0.45	6.00	0.43	0.75	0.11	0.18	11.00	0.46	0.70	0.01	− 0.08
Uganda (*n* = 63)	5.00	0.14	0.73	0.04	0.68	7.25	0.46	0.82	0.04	0.31	9.50	0.66	0.83	0.02	0.10	8.00	0.51	0.85	0.01	0.35
Zambia (*n* = 60)	3.00	0.00	0.65	0.00	0.82	9.00	0.37	0.81	0.01	0.49	6.50	0.43	0.80	0.00	0.25	13.50	0.52	0.86	− 0.01	0.28
Zimbabwe (*n* = 70)	6.50	0.41	0.66	0.03	− 0.36	6.00	0.33	0.72	0.09	0.22	7.00	0.37	0.76	0.03	0.19	9.50	0.28	0.65	− 0.02	0.42

Locus	AFUB12 (3L)					AFND19 (3R)					AFND20 (3R)					AFNB41 (3R)				
Malawi (*n* = 60)	11.67	0.36	0.89	0.00	0.57	3.00	0.00	0.61	0.10	0.78	11.33	0.36	0.87	− 0.01	0.56	9.67	0.20	0.87	0.01	0.73
Mozambique (*n* = 70)	8.50	0.63	0.76	0.17	0.16	3.50	0.13	0.64	0.15	0.71	7.00	0.74	0.76	− 0.02	1.00	3.50	0.00	0.34	− 0.01	− 0.18
Uganda (*n* = 63)	10.25	0.45	0.88	0.02	0.43	3.50	0.30	0.61	0.23	0.06	8.25	0.16	0.85	0.00	0.78	11.00	0.49	0.91	0.00	0.39
Zambia (*n* =60)	11.00	0.47	0.84	0.12	0.36	3.00	0.22	0.50	0.02	− 0.17	8.00	0.17	0.79	0.03	0.83	9.00	0.45	0.84	0.00	0.28
Zimbabwe (*n* = 70)	11.00	0.39	0.84	− 0.02	0.36	7.50	0.16	0.82	0.08	0.71	7.00	0.23	0.82	0.01	0.62	na	na	na	na	na

*Abbreviations*: Na, allelic richness; Ho, observed heterozygosity; He, expected heterozygosity; F_ST_, fixation index; F_IS_, inbreeding coefficient; na, not applicable

### Hardy–Weinberg equilibrium

When samples were analyzed as a single population (i.e. pooled together), three loci (AFUB19, FUNQ and FUNF) of the twelve showed significant deviations from the Hardy-Weinberg equilibrium (HWE). This may have been due to significant heterozygote deficiency or due to the sub-structuring within the population. When these were removed no significant deviation was observed.

### Genetic differentiation and population structure

Genetic divergence between the sampling groups estimated by F_ST_ ranged from low to moderate. Values between 0.00–0.05 indicate little divergence, 0.05–0.15 moderate divergence, 0.15–0.25 high divergence and over 0.25 a very high degree of divergence [30, 38, 39]. Genetic differentiation between all pairs of the sites and samples was estimated based on allele frequency differences at microsatellite level between the studied populations. The values of F_ST_ between pairwise population comparisons for all loci varied from 0.006 to 0.396 across populations. These were significant within countries (*P* < 0.05) for Mozambique (Maciana and Matola), Uganda (between Kamuli and the other three localities) and Zambia (Nchelenge and Namwala) (Table 4). The highest pairwise F_ST_ estimates were obtained for the Malawi sites (Likoma and Majete) and the Zimbabwe sites (Honde and Mangwanda) but these were not significantly different (*P* > 0.05). Sites in Malawi varied between 0.029 (Likoma) to 0.054 (Karonga), 0.12 between Maciana and Matola, 0.013 between Mangwanda and Honde and the highest divergence was between all the sites in northern Uganda and Kamuli. Over all populations studied, the F_ST_ estimate was 0.116 indicating that there was some genetic structure in the dataset (*P* < 0.05). This suggests that there are levels of genetic differentiation among populations within the selected countries.

**Table 4:** Pairwise comparison of genetic diversity (FST) among the 13 geographical *An. funestus* populations sampled

	Malawi			Mozambique		Zambia		Zimbabwe		Uganda			
	Kar	Lik	Maj	Mac	Mat	Nche	Nam	Man	Hon	Lira	Agul	Apa	Kam
Kar	0.000												
Lik	0.044	0.000											
Maj	0.054	0.029	0.000										
Mac	− 0.022	0.007	0.014	0.000									
Mat	− 0.017	− 0.073	0.038	0.109*	0.000								
Nche	− 0.033	0.045	0.025	0.014	0.038	0.000							
Nam	0.093	0.092	0.108	0.120	0.025	0.032*	0.000						
Man	0.034	− 0.027	− 0.040	0.029	0.025	0.093	0.108	0.000					
Hon	0.092	− 0.034	− 0.027	− 0.019	0.040	0.009	0.012	0.396	0.000				
Lira	0.022	0.007	0.013	0.041	0.126	0.063	0.014	0.053	0.170	0.000			
Agul	0.016	0.021	0.018	0.210	0.247	0.038	0.026	0.016	0.012	0.023	0.000		
Apa	0.034	0.052	0.127	0.019	0.018	0.041	0.013	0.035	0.198	0.029	0.026	0.000	
Kam	0.112	0.191	0.135	0.891	0.198	0.135	0.298	0.144	0.112	0.054*	0.006*	0.070*	0.000

*P < 0.05

*Abbreviations*: Mal, Malawi; Kar, Karonga; Lik, Likoma; Maj, Majete; Moz, Mozambique; Mac, Maciana; Mat, Matola; Zam, Zambia; Nche, Nchelenge; Nam, Namwala; Zim, Zimbabwe; Man, Mangwanda; Hon, Honde; Uga, Uganda; Lir, Lira; Agul, Agule; Apa, Apac; Kam, Kamuli

**Fig. 2:**
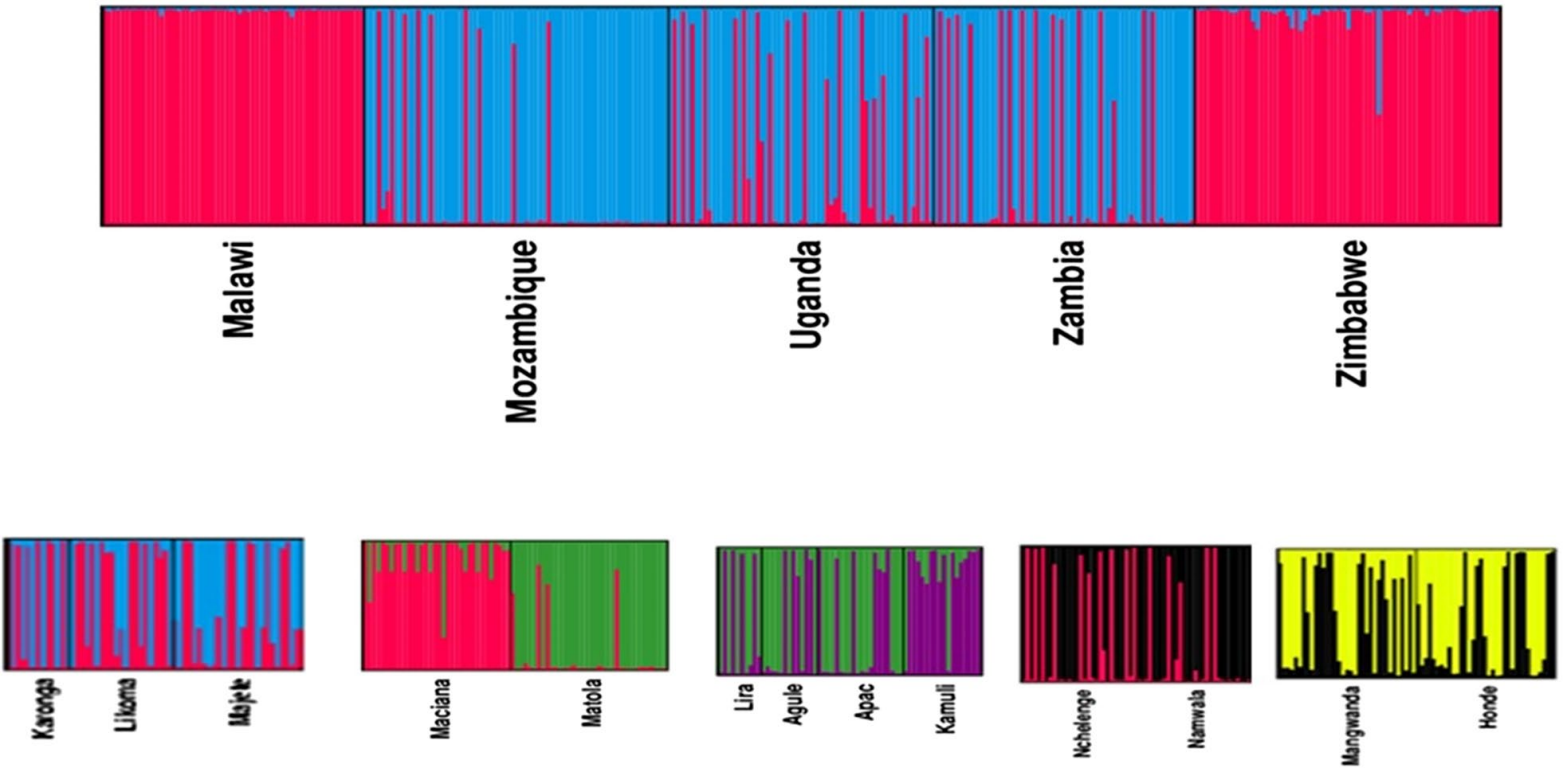
Bayesian cluster analysis using STRUCTURE. Graphical representation of the data set for the most likely K (K = 2), where each colour corresponds to a suggested cluster and each individual is represented by a vertical bar. The X-axis corresponds to the population codes. The Y-axis presents the probability of assignment of an individual to each cluster. Above are clusters for all five countries and beneath are the clusters for each of the 13 sites

STRUCTURE provided consistent results over 5 replicated runs tested for each K. The probability of data Ln Pr (X|K) increased from K = 1 to K = 2 until it reached a maximum value at K = 2, after which values decreased gradually for all countries (Fig. 2). Thus, in agreement with the F_ST_ results, the most likely number of genetic clusters in the dataset was two. However, when STRUCTURE was re-run with only 8 loci, three clusters were obtained for Malawi and Mozambique, and the rest of the countries retained the original two clusters (Additional file 2: Figure S1).

Additional insight into how variation was partitioned across the countries was shown by an analysis of molecular variance (AMOVA). The AMOVA of all twelve microsatellites confirmed the differentiation and structure analysis with the variation between individuals, within populations, and among populations, being 38%, 50.24% and 0.21% (Table 5), respectively. Variation among individuals within populations and among individuals explained 50% and 38% of the total variation, respectively. Almost all genetic diversity (88%) was partitioned within populations, indicating small differences. Global F_ST_ estimates revealed a slight but significant overall degree of genetic divergence (*P* < 0.05 on 30,000 Markov chains) (Table 6). Only for Uganda were conditions suitable for AMOVA and much of the variation was recorded as among individuals within populations. This is in accordance with other studies that have found that most of the variation was with individuals among populations [12, 40–42].

**Table 5:** Analysis of molecular variance (AMOVA) of twelve microsatellite loci in the *An. funestus* populations from Malawi, Mozambique, Uganda, Zambia and Zimbabwe

Source of variation	Sum of squares	Variance components	Percentage variation
Among groups	123.335	0.008	0.214
Among groups within populations	121.684	0.429	11.074
Among individuals within populations	1701.694	1.947	50.247
Within individuals	478.500	1.491	38.466

*Note*: Most of the variation is among individuals within the selected populations

**Table 6:** Global test of differentiation

Country	Malawi	Mozambique	Uganda	Zambia	Zimbabwe
Malawi	0.000	0.000 ± 0.000	0.000 ± 0.000	0.000 ± 0.000	0.000 ± 0.000
Mozambique	0.116*	0.000	0.000 ± 0.000	0.000 ± 0.000	0.000 ± 0.000
Uganda	0.125*	0.135*	0.000	0.009 ± 0.009	0.000 ± 0.000
Zambia	0.141*	0.141*	0.028*	0.000	0.000 ± 0.000
Zimbabwe	0.094*	0.136*	0.116*	0.117*	0.000

*P < 0.05

### Isolation by distance and gene flow

A Mantel test for Matrix correspondence called the isolation by distance (IBD) hypothesis was performed separately for each country using GenAlEx 6.501 to test for the occurrence of a positive correlation (Rxy > 0) between the genetic matrix and the geographical distances. For Malawi, the villages of Karonga, Likoma and Majete showed an Rxy of 0.083, *P* = 0.007; for Mozambique, Maciana and Matola villages, Rxy = 0.257, *P* = 0.07; for Zambia, Nchelenge and Namwala, Rxy = 0.026, *P* = 0.007; for Zimbabwe, Mangwanda and Honde Rxy 0.071, *P* = 0.005; and for Uganda, Kamuli, Apac, Agule and Lira, Rxy = 0.15, *P* = 0.023. A low but significant correlation for Malawi, Zambia, Zimbabwe and Uganda was observed. No significant difference was observed for Mozambique (*P* < 0.05). The estimates of gene flow among *An. funestus* populations suggests that there is a range of gene flow between country level collections (N_em_ ranging from 0.03 to 41), with some populations more reproductively isolated than others with N_em_ country averages of 6, 2, 8, 18 and 14, for Malawi, Mozambique, Zambia, Zimbabwe and Uganda, respectively (Table 7). The highest N_em_ country average was in the Zimbabwe populations.

**Table 7:** N_em_ comparisons for all the five countries

	Malawi			Mozambique		Zambia		Zimbabwe		Uganda			
	Kar	Lik	Maj	Mac	Mat	Nche	Nam	Man	Hon	Lira	Agul	Apa	Kam
Kar	–												
Lik	5.43	–											
Maj	4.38	8.37	–										
Mac	11.36	35.46	2.1	–									
Mat	14.45	3.17	6.32	2.04	–								
Nche	7.33	5.30	9.75	17.60	6.32	–							
Nam	2.44	2.47	2.06	1.83	9.75	6.81	–						
Man	7.35	9.01	6.00	8.37	9.75	2.44	2.06	–					
Hon	2.47	7.10	9.01	13.15	6.00	27.52	20.83	18.2	–				
Lira	11.36	35.46	18.98	5.85	1.73	3.72	17.60	4.47	1.22	–			
Agul	17.60	11.65	13.64	0.94	0.76	6.33	9.34	15.38	20.58	10.86	–		
Apa	7.10	4.56	1.72	12.90	13.64	5.85	18.98	6.89	1.01	8.37	9.36	–	
Kam	1.98	1.06	1.60	0.03	1.01	1.60	0.59	1.49	1.98	5.43	41.4	1.54	–

*Abbreviations*: Mal; Malawi; Kar, Karonga; Lik, Likoma; Maj, Majete; Moz, Mozambique; Mac, Maciana; Mat, Matola; Zam, Zambia; Nche, Nchelenge; Nam, Namwala; Zim, Zimbabwe; Man, Mangwanda; Hon, Honde; Uga, Uganda; Lir, Lira; Agul, Agule; Apa, Apac; Kam, Kamuli

### Principal coordinates analysis

**Fig. 3:**
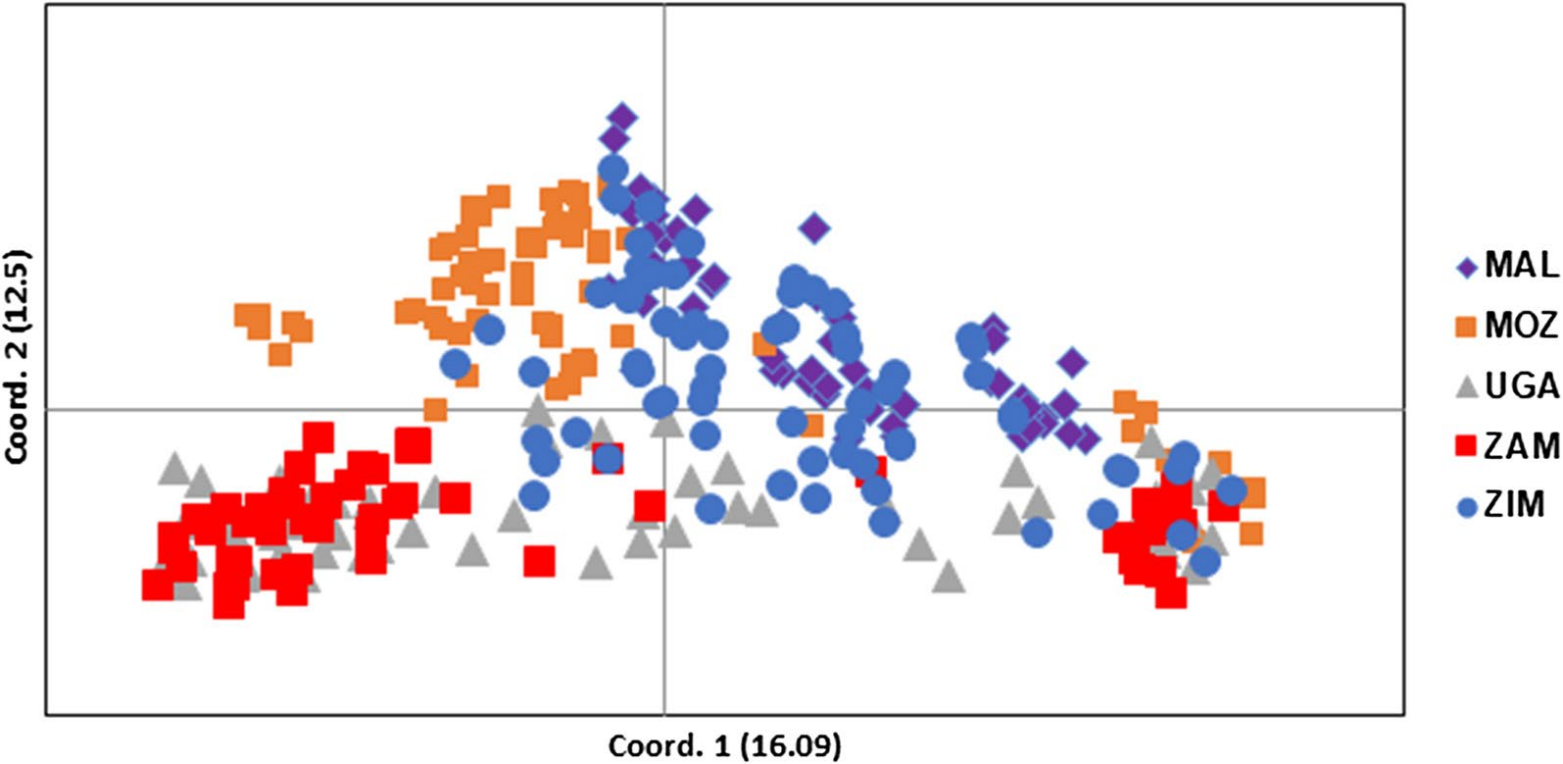
Principal coordinates analysis (PCoA) generated from 12 microsatellites among five *An. funestus* populations from Uganda and southern African countries. *Abbreviations*: MAL, Malawi; MOZ, Mozambique; UGA, Uganda; ZAM, Zambia; ZIM, Zimbabwe

The PCoA analysis found that the two most important principal coordinate axes accounted for less than 80% of the total variance in relatedness between genetic and geographical distance for *An*. *funestus* microsatellite loci, which would be the threshold required for statistical significance in PCoA (Fig. 3). The top two PCoA components explained 16.1% and 12.5% of the total variance and grouped the individuals into two main clusters (Fig. 3). Groupings were between Malawi and Mozambique, and Uganda and Zambia. Zimbabwe samples uniquely mixed with all the other populations.

## Discussion

Genetic methods of assessing *An. funestus* population structure and differentiation were employed to provide information on within and between population differentiation, diversity and gene flow. Understanding these characteristics allows for better tailoring of vector control methods. Molecular data revealed overall high genetic variability among *An. funestus* populations from eastern and southern Africa, highlighting an important level of allelic richness and gene diversity.

Low to moderate differentiation of the 13 *An. funestus* populations studied was shown at the nuclear DNA level with the 12 microsatellites genotyping as polymorphic across sites. This is consistent with low to moderate levels of genetic differentiation found in individuals among *An. funestus* populations in east, west and southern Africa [12, 18, 19, 39–42] and in other *Anopheles* species [19, 43–45]. The low to moderate levels of genetic differentiation may also be the result of barriers to gene flow between the populations. In our case, genetic differentiation among the sampled *An. funestus* populations had a weak yet significant correlation with geographical distance and therefore might be affected by other factors that need investigation. Our findings confirm those of Ayala et al. [40], Michel et al. [12] and Barnes et al. [15], who found *An. funestus* to be structured following a similar pattern of isolation by distance. The differentiation deviations could be a result of inbreeding as observed in the high F_IS_ values for some populations. In addition, these values could also be attributed to the presence of the Wahlund effect (reduction of heterozygosity caused by subpopulation structure) or the spatial pooling of individuals originating from different houses or focal points [46]. Changes have taken place over the studied populations and collections were done at different times and over different years and this could be influencing the HWE of *An. funestus* populations [47, 48]. HWE states that the genes and genotypes will remain constant in the absence of migration, genetic drift and inbreeding from generation to generation [30, 48]. The changes in this instance could also have been caused by epistatic natural selection and random genetic drift. These features can affect the flow of genes conditioning vector competence and insecticide resistance. However, other factors such as microclimate, increasing urbanization and global warming may play a part that needs to be investigated.

The rate of gene flow over time is considered suitable for estimating exchange of genes between populations [38]. N_*em*_ values of gene flow for *An. funestus* populations have been reported to vary from 17 to 483 [12, 18, 39]. The recorded values of N_*em*_ for *An. funestus* in this study were much lower [3–40], probably as a result of the geographical distances between the populations which ranged from 140 km between Maciana and Matola in Mozambique, to 3000 km between the most southern site of Matola and the most northern site of Lira in Uganda. Furthermore, samples were collected at different time points spanning 10 years and in different seasons. Thus geographical distances that separate the studied populations may not be the only barriers to limit gene flow among them, but other environmental factors may also play a role. Analysis of molecular variance indicated that most of the genetic variation (88%) was maintained within the populations rather than between them. These results confirm findings of Barnes et al. [15], Michel et al. [12] and Ogola et al. [18] from countries in east, west and southern Africa.

A study on upregulation of insecticide resistance metabolic enzyme genes provided evidence of population structuring between northern and southern sites in Malawi [16]. The Mantel test used here for correlation of genetic variation with geographical distance (isolation by distance hypothesis) showed positive correlation between sites in Malawi (*R*_*xy*_ = 0.083, *P* = 0.007), not indicating environmental or other barriers to gene flow. Only the two sites in Mozambique (Matola and Maciana, 140 km apart) gave negative results for isolation by distance. Genetic/geographical distance correlations are variable with previous studies showing these to be weak but significant [12, 22], weak and non-significant [18], or non-existent [19].

Using genetic structure analysis, the populations studied here were clustered into two groups, Malawi/Zimbabwe *versus* Uganda/Zambia/Mozambique. These groups do not coincide with the mitochondrial Clades I and II of Choi et al. [21], both of which were shown to occur in Mozambique and Zambia in the present study. A recent study by Jones et al. [37] based on the full mitogenomes of *An. funestus* from Zambia, the Democratic Republic of Congo and Tanzania, showed that there were two strong maternal lineages in Zambia and Tanzania. Whether the PCoA results illustrated in Fig. 3 here, showing two distinct clusters in the Zambian sample reflect the mitogenome clusters, remains to be confirmed. The TaqMan assay [21] was not always accurate in identifying the clades [37] and this might also impact on the results shown in our study.

## Conclusions

Two genetically distinct clusters were revealed in *An. funestus* with populations from Mozambique, Uganda and Zambia forming one group and Malawi and Zimbabwe the second group. However, almost all genetic diversity (88%) was partitioned within populations, indicating small differences, except for the PCoA of the Zambian samples which were clearly divergent. The variable effective population sizes, climate and different collection time points, may be some of the factors affecting the differentiation. Future research should investigate changes in mosquito populations over time, especially as insecticide use and their coverage evolve, new interventions are rolled out, and climate/land use changes.

## Supplementary information

**Additional file 1: Table S1.** Population comparisons, HW proportions and independence of loci (significant values recorded in bold).

**Additional file 2: Figure S1**. Structure clusters by population in each of the countries, Malawi, Mozambique, Uganda, Zambia and Zimbabwe with eight microsatellites. The X-axis corresponds to the population codes. The Y-axis presents the probability of assignment of a population to each cluster.

## Data Availability

All data generated or analysed during this study are included in this published article and its additional files.

## Abbreviations

PCR: Polymerase chain reaction
AMOVA: Analysis of molecular variance
FSTAT: Fisherʼs exact test
IBD: Isolation by distance
MCMC: Markov Chain Monte Carlo
PP: Primers present in analysis column
IAC: Indoor aspiration collection
HLC: Human landing collection
*s.s.*: *Sensu stricto*

## References

[1] Gillies MT, De Meillon B (1968). The Anophelinae of Africa South of the Sahara. Publ S Afr Inst Med Res.

[2] Gillies MT, Coetzee M (1987). A supplement to the Anophelinae of Africa South of the Sahara. Publ S Afr Inst Med Res.

[3] Sinka ME, Bangs MJ, Manguin S, Coetzee M, Mbogo CM, Hemingway J (2010). The dominant *Anopheles* vectors of human malaria in Africa, Europe and the Middle East: occurrence data, distribution maps and bionomic précis. Parasites Vectors.

[4] Coetzee M, Fontenille D (2004). Advances in the study of *Anopheles funestus*, a major vector of malaria in Africa. Insect Biochem Mol Biol.

[5] Coetzee M, Koekemoer LL (2013). Molecular systematics and insecticide resistance in the major African malaria vector, *Anopheles funestus*. Ann Rev Entomol.

[6] Choi KS, Christian R, Nardini L, Wood OR, Agubuzo E, Muleba M (2014). Insecticide resistance and role in malaria transmission of *Anopheles funestus* populations from Zambia and Zimbabwe. Parasites Vectors.

[7] Hargreaves K, Koekemoer LL, Brooke BD, Hunt RH, Mthembu J, Coetzee M (2000). *Anopheles funestus* is resistant to pyrethroid insecticides in South Africa. Med Vet Entomol.

[8] Mugenzi LMJ, Menze BD, Tchouakui M, Wondji MJ, Irving H, Tchoupo M (2019). Cis-regulatory CYP6P9b P450 variants associated with loss of insecticide-treated bed net efficacy against *Anopheles funestus*. Nat Commun.

[9] Riveron JM, Huijben S, Tchapga W, Tchouakui M, Wondji MJ, Tchoupo M (2019). Escalation of pyrethroid resistance in the malaria vector *Anopheles funestus* induces a loss of efficacy of piperonyl butoxide-based insecticide-treated nets in Mozambique. J Infect Dis.

[10] WHO. World malaria report 2018. Geneva: World Health Organization; 2018. https://www.who.int/malaria/publications/world-malaria-report-2018/en. Accessed 28 Jan 2019.

[11] Sinka ME, Golding N, Massey NC, Wiebe A, Huang Z, Hay SI, Moyes CL (2016). Modelling the relative abundance of the primary African vectors of malaria before and after the implementation of indoor, insecticide-based vector control. Malar J.

[12] Michel AP, Ingrasci MJ, Schemerhorn BJ, Kern M, Le Goff G, Coetzee M (2005). Rangewide population genetic structure of the African malaria vector *Anopheles funestus*. Mol Ecol.

[13] Morgan JC, Irving H, Okedi LM, Steven A, Wondji CS (2010). Pyrethroid resistance in an *Anopheles funestus* population from Uganda. PLoS ONE.

[14] Mulamba C, Riveron JM, Ibrahim SS, Irving H, Barnes KG, Mukwaya LG (2014). Widespread pyrethroid and DDT resistance in the major malaria vector *Anopheles funestus* in East Africa is driven by metabolic resistance mechanisms. PLoS ONE.

[15] Barnes KG, Weedall GD, Ndula M, Irving H, Mzihalowa T, Hemingway J, Wondji CS (2017). Genomic footprints of selective sweeps from metabolic resistance to pyrethroids in African malaria vectors are driven by scale up of insecticide-based vector control. PLoS Genet.

[16] Barnes KG, Irving H, Chiumia M, Mzilahowa T, Coleman M, Hemingway J, Wondji CS (2017). Restriction to gene flow is associated with changes in the molecular basis of pyrethroid resistance in the malaria vector *Anopheles funestus*. Proc Nat Acad Sci USA.

[17] Choi KS, Koekemoer LL, Coetzee M (2012). Population genetic structure of the major malaria vector *Anopheles funestus s.s.* and allied species in southern Africa. Parasites Vectors.

[18] Ogola EO, Odero JO, Mwangangi JM, Masiga DK, Tchouassi DP (2019). Population genetics of *Anopheles funestus*, the African malaria vector, Kenya. Parasites Vectors.

[19] Gélin P, Magalon H, Drakeley C, Maxwell C, Magesa S, Takken W, Boëte C (2016). The fine-scale genetic structure of the malaria vectors *Anopheles funestus* and *Anopheles gambiae* (Diptera: Culicidae) in the north-eastern part of Tanzania. Int J Trop Insect Sci.

[20] Koekemoer LL, Kamau L, Hunt RH, Coetzee M (2002). A cocktail polymerase chain reaction assay to identify members of the *Anopheles funestus* (Diptera: Culicidae) group. Am J Trop Med Hyg.

[21] Choi KS, Coetzee M, Koekemoer LL (2013). Detection of clade types (clades I and II) within *Anopheles funestus sensu stricto* by the hydrolysis probe analysis (TaqMan assay). Parasites Vectors.

[22] Cohuet A, Simard F, Berthomieu A, Raymond M, Fontenille D, Weill M (2002). Isolation and characterization of microsatellite DNA markers in the malaria vector *Anopheles funestus*. Mol Ecol Notes.

[23] Sharakov IV, Braginets O, Mbogo CN, Yan G (2001). Isolation and characterization of trinucleotide microsatellites in the African malaria mosquito *Anopheles funestus*. Mol Ecol Notes.

[24] Sharakov I, Braginets O, Grushko O, Cohuet A, Guebeogo M, Boccolini D (2004). A microsatellite map of the African human malaria vector *Anopheles funestus*. J Hered.

[25] Schermerhorn BJ, Greeman S, Banks M, Vulule J, Sagnon N’F, Costantini C, Besansky NJ (2003). Dinucleotide microsatellite markers from *Anopheles funestus*. Mol Ecol Notes.

[26] Excoffier L, Lischer HEL (2010). Arlequin suite version 3.5: a new series of programs to perform population genetics analyses under Linux and Windows. Mol Ecol Resour.

[27] Goudet J. FSTAT, Version 2.9.3. A program to estimate and test gene diversities and fixation indices. 2001. www.unil.ch/izea/softwares/fstat.html. Accessed 17 Jan 2017.

[28] Weir BS, Cockerham CC (1984). Estimating F-statistics for the analysis of population structure. Evolution.

[29] Excoffier L, Smouse PE, Quattro JM (1992). Analysis of molecular variance inferred from metric distances among DNA haplotypes: application to human mitochondrial DNA restriction data. Genetics.

[30] Pritchard JK, Stephens M, Donnelly P (2000). Inference of population structure using multilocus genotype data. Genetics.

[31] Earl DA, von Holdt BM (2012). STRUCTURE HARVESTER: a website and program for visualizing STRUCTURE output and implementing the Evanno method. Conserv Genet Resour.

[32] Jakobsson M, Rosenberg NA (2007). CLUMPP: a cluster matching and permutation program for dealing with label switching and multimodality in analysis of population structure. Bioinformatics.

[33] Rosenberg NA (2004). Distruct: a program for the graphical display of population structure. Mol Ecol Notes.

[34] Slatkin M (1995). A measure of population subdivision based on microsatellite allele frequencies. Genetics.

[35] Peakall R, Smouse PE (2006). GENALEX 6: genetic analysis in Excel. Population genetic software for teaching and research. Mol Ecol Notes.

[36] Peakall R, Smouse PE (2012). GenAlEx 6.5: genetic analysis in Excel. Population genetic software for teaching and research-an update. Bioinformatics.

[37] Jones CM, Lee Y, Kitchen A, Collier T, Pringle JC, Muleba M (2018). Complete *Anopheles funestus* mitogenomes reveal an ancient history of mitochondrial lineages and their distribution in southern and central Africa. Sci Rep.

[38] Slatkin S (1985). Rare alleles as indicators of gene flow. Evolution.

[39] Braginets OP, Minakawa N, Mbogo CM, Yan G (2003). Population genetic structure of the African malaria mosquito *Anopheles funestus* in Kenya. Am J Trop Med Hyg.

[40] Ayala D, Le Goff G, Robert V, Takken WJ (2006). Population structure of the malaria vector *Anopheles funestus* (Diptera: Culicidae) in Madagascar and Comoros. Acta Trop.

[41] Samb B, Dia I, Konate L, Ayala D, Fontenille D, Cohuet A (2012). Population genetic structure of the malaria vector *Anopheles funestus*, in a recently re-colonized area of the Senegal River basin and human-induced environmental changes. Parasites Vectors.

[42] Michel AP, Grushko O, Wamdaogo M, Guelbeogo WM, Lobo NF, Sagnon N (2006). Divergence with gene flow in *Anopheles funestus* from the Sudan savanna of Burkina Faso, West Africa. Genetics.

[43] Lehmann T, Licht M, Elissa N, Maega BT, Chimumbwa JM, Watsenga FT (2003). Population structure of *Anopheles gambiae* in Africa. J Hered.

[44] Lehmann T, Besansky NJ, Hawley WA, Fahey TG, Kamau L, Collins FH (1997). Microgeographic structure of *Anopheles gambiae* in western Kenya based on mtDNA and microsatellite loci. Mol Ecol.

[45] Antonio-Nkondjio C, Ndo C, Kengne P, Mukwaya L, Awono-Ambene P, Fontenille D, Simard F (2008). Population structure of the malaria vector *Anopheles moucheti* in the equatorial forest region of Africa. Malar J.

[46] Carja O, Liberman U, Feldman MW (2014). Evolution in changing environments: modifiers of mutation, recombination and migration. Proc Nat Acad Sci.

[47] Ma L, Zhang D, Ji YJ (2015). Statistical measures of genetic differentiation of populations: rationales, history and current states. Curr Zool.

[48] Bacaer N, Bacaer N (2011). The Hardy–Weinberg law (1908). A short history of mathematical population dynamics.

