# Supplementary material for: Genetic differentiation and population structure of Anopheles funestus from Uganda and the southern African countries of Malawi, Mozambique, Zambia and Zimbabwe
Source: Parasit Vectors. 2020 Feb 18;13:87. doi: 10.1186/s13071-020-3962-1 (PMC7029513; doi:10.1186/s13071-020-3962-1)
Supplement: Supplementary file 1 — Additional file 1: Table S1. Population comparisons, HW proportions and independence of loci (significant values recorded in bold). [file 13071_2020_3962_MOESM1_ESM.docx]

**Additional file 1: Table S1.** Population comparisons, HW proportions and independence of loci (significant values recorded in bold)

| **Country** | **Site / Locus** | **FUNL N_a_** | **(2L) Ho** | **H_E_** | **F_ST_** | **F_IS_** | **FUNO N_a_** | **(2R) Ho** | **H_E_** | **F_ST_** | **F_IS_** | **FUNQ N_a_** | **(X) Ho** | **H_E_** | **F_ST_** | **F_IS_** | **FUNF N_a_** | **(2R) Ho** | **H_E_** | **F_ST_** | **F_IS_** |
| --- | --- | --- | --- | --- | --- | --- | --- | --- | --- | --- | --- | --- | --- | --- | --- | --- | --- | --- | --- | --- | --- |
| **Malawi** (n=  60) | Karonga (n =13) | 6 | 0.615 | 0.831 | 0.123 | 0.178 | 4 | 0.923 | 0.699 | 0.033 | 0.271 | 3 | 0 | 0.69 | 0.2 | 1 | 4 | 0.615 | 0.67 | -0 | 0.1 |
|  | Majete (n = 26) | 7 | 0.077 | 0.851 | 0.11 | 0.242 | 4 | 0.192 | 0.661 | 0.083 | -0.58 | 6 | 0.23 | 0.8 | 0.2 | 1 | 5 | 0.385 | 0.72 | -0 | -0.3 |
|  | Likoma (n = 21) | 8 | 0.429 | 0.768 | 0.168 | 0.896 | 8 | 0.285 | 0.832 | 0.011 | 0.638 | 5 | 0.14 | 0.79 | 0.2 | 1 | 5 | 0.476 | 0.71 | -0 | 0.1 |
| **Mozambique** (n = 70) | Maciana (n = 34) | 8 | 0.324 | 0.831 | 0.029 | 0.444 | 5 | 0.5 | 0.696 | 0.249 | 0.35 | 3 | 0.38 | 0.57 | 0.06 | 0.3 | 3 | 0.235 | 0.65 | -0 | 0.4 |
|  | Matola (n = 36) | 7 | 0.889 | 0.818 | 0.003 | -0.09 | 6 | 0.278 | 0.714 | 0.254 | 0.549 | 3 | 0.31 | 0.55 | 0.06 | **0.4** | 3 | 0.222 | 0.66 | 0.34 | 0.4 |
| **Uganda**  (n = 63) | Agule (n = 13) | 4 | 0.615 | 0.735 | 0.106 | -0.45 | 5 | 0.231 | 0.739 | 0.018 | 0.239 | 3 | 0 | 0.64 | 0.17 | 1 | 5 | 0.077 | 0.73 | 0.1 | 0.8 |
|  | Lira (n = 12) | 5 | 0.7 | 0.784 | 0.44 | -0.58 | 7 | 0.5 | 0.847 | 0.068 | 0.484 | 2 | 0 | 0.51 | 0.04 | 1 | 5 | 0.1 | 0.74 | -0 | 0.9 |
|  | Apac (n = 18) | 10 | 0.65 | 0.819 | -0.024 | 0.014 | 10 | 0.65 | 0.864 | 0.026 | 0.145 | 3 | 0 | 0.68 | 0.09 | 1 | 3 | 0 | 0.67 | 0.05 | **0.9** |
|  | Kamuli (n = 20) | 6 | 0.444 | 0.705 | -0.02 | 0.125 | 7 | 0.444 | 0.819 | 0.038 | 0.368 | 5 | 0 | 0.69 | 0.11 | 1 | 7 | 0.369 | 0.78 | 0.01 | 0.2 |
| **Zambia**  (n =60) | Nchelenge (n =30) | 12 | 0.533 | 0.855 | -0.025 | 0.26 | 10 | 0.467 | 0.828 | -0.028 | 0.413 | 3 | 0.27 | 0.6 | 0 | 0.8 | 3 | 0 | 0.65 | 0 | 0.8 |
|  | Namwala (n = 30) | 12 | 0.633 | 0.866 | -0.019 | 0.169 | 8 | 0.267 | 0.798 | 0.056 | 0.575 | 3 | 0.07 | 0.63 | 0 | 0.8 | 3 | 0 | 0.64 | 0 | 0.8 |
| **Zimbabwe** (n=70) | Mangwanda (n= 35) | 4 | 0.242 | 0.669 | -0.022 | 0.27 | 6 | 0.364 | 0.711 | 0.082 | 0.099 | 7 | 0.12 | 0.83 | 0.09 | 1 | 9 | 0.424 | 0.66 | 0.06 | -0.3 |
|  | Honde (n = 35) | 4 | 0.216 | 0.662 | -0.008 | 0.506 | 6 | 0.297 | 0.735 | 0.105 | 0.337 | 3 | 0 | 0.64 | 0.34 | 0.9 | 4 | 0.405 | 0.66 | -0 | -0.4 |

**Table S1 cont’d:** Population comparisons, HW proportions and independence of loci (significant values recorded in bold)

| **Country** | **Site / Locus** | **AFUB3 N_a_** | **(2R) Ho** | **H_E_** | **F_ST_** | **F_IS_** | **AFUB10 N_a_** | **(2L) Ho** | **H_E_** | **F_ST_** | **F_IS_** | **AFUB11 N_a_** | **(2L) Ho** | **H_E_** | **F_ST_** | **F_IS_** | **AFUB12 N_a_** | **(3L) Ho** | **H_E_** | **F_ST_** | **F_IS_** |
| --- | --- | --- | --- | --- | --- | --- | --- | --- | --- | --- | --- | --- | --- | --- | --- | --- | --- | --- | --- | --- | --- |
| **Malawi** (n= 60) | Karonga (n =13) | 4 | 0.923 | 0.689 | -0.002 | -0.87 | 8 | 0.23 | 0.855 | 0.021 | 0.74 | 6 | 0 | 0.8 | -0.06 | 1 | 11 | 0.39 | 0.9 | 0.035 | 0.567 |
|  | Majete (n = 26) | 6 | 0.962 | 0.711 | -0.023 | -0.61 | 9 | 0.35 | 0.805 | -0.04 | 0.71 | 6 | 0 | 0.8 | -0.06 | 1 | 14 | 0.42 | 0.9 | -0.01 | **0.623** |
|  | Likoma (n = 21) | 6 | 0.952 | **0.731** | -0.023 | -0.44 | 9 | 0.24 | 0.857 | -0.04 | 0.58 | 6 | 0 | 0.8 | -0.06 | **1** | 10 | 0.29 | 0.9 | -0.02 | 0.507 |
| **Mozambique**  (n = 70) | Maciana (n = 34) | 12 | 0.559 | 0.749 | 0.02 | -0.47 | 4 | 0.62 | 0.741 | 0.007 | -0.31 | - | - | - | - | - | 10 | 0.68 | 0.8 | 0.152 | 0.137 |
|  | Matola  (n = 36) | 10 | 0.361 | 0.66 | 0.002 | 0.306 | 4 | 0.58 | 0.758 | 0.003 | -0.11 | - | - | - | - | - | 7 | 0.58 | 0.7 | 0.191 | 0.177 |
| **Uganda** (n = 63) | Agule  (n = 13) | 10 | 0.462 | 0.892 | 0.033 | 0.16 | 5 | 0.08 | 0.6 | 0.028 | 0.72 | 5 | 0.15 | 0.7 | 0.016 | 0.6 | 10 | 0.31 | 0.9 | 0.018 | 0.492 |
|  | Lira  (n = 12) | 6 | 0.7 | 0.826 | 0.003 | 0.341 | 4 | 0 | 0.758 | -0.04 | 0.77 | 4 | 0.2 | 0.7 | 0.065 | 0.7 | 9 | 0.4 | 0.9 | 0.023 | 0.579 |
|  | Apac  (n = 18) | 7 | 0.2 | 0.831 | 0.018 | **0.674** | 8 | 0.05 | 0.755 | -0.02 | 0.9 | 4 | 0.1 | 0.4 | -0.02 | 0.6 | 10 | 0.6 | 0.9 | 0.008 | 0.25 |
|  | Kamuli  (n = 20) | 9 | 0.667 | 0.86 | -0.011 | 0.23 | 6 | 0.06 | 0.76 | -0.05 | 0.9 | 4 | 0.06 | 0.7 | 0.007 | 0.9 | 12 | 0.5 | 0.9 | 0.011 | 0.395 |
| **Zambia** (n =60) | Nchelenge (n =30) | 13 | 0.467 | 0.843 | -0.022 | 0.24 | - | - | - | - | - | 19 | 0.53 | 0.9 | 0.057 | 0.4 | 13 | 0.53 | 0.9 | 0.067 | 0.313 |
|  | Namwala (n = 30) | 14 | 0.567 | 0.87 | 0.002 | 0.323 | - | - | - | - | - | 14 | 0.43 | 0.9 | 0.022 | 0.4 | 9 | 0.4 | 0.8 | 0.167 | 0.412 |
| **Zimbabwe** (n=70) | Mangwanda (n = 35) | 10 | 0.273 | 0.658 | -0.027 | 0.423 | 6 | 0.3 | 0.744 | 0.018 | 0.34 | 14 | 0.49 | 0.9 | 0.008 | 0.4 | 12 | 0.42 | 0.8 | -0.02 | 0.337 |
|  | Honde  (n = 35) | 9 | 0.279 | 0.636 | -0.021 | 0.419 | 6 | 0 | 0.717 | 0.001 | 1 | 12 | 0.27 | 0.8 | 0.026 | 0.7 | 10 | 0.35 | 0.8 | -0.02 | 0.38 |

**Table S1 cont’d:** Population comparisons, HW proportions and independence of loci (significant values recorded in bold)

| **Country** | **Site / Locus** | **AFND19 N_a_** | **(3R) Ho** | **H_E_** | **F_ST_** | **F_IS_** | **AFND20 N_a_** | **(3R) Ho** | **H_E_** | **F_ST_** | **F_IS_** | **AFND23 N_a_** | **(2R) Ho** | **H_E_** | **F_ST_** | **F_IS_** | **AFNB41 N_a_** | **(3R) Ho** | **H_E_** | **F_ST_** | **F_IS_** |
| --- | --- | --- | --- | --- | --- | --- | --- | --- | --- | --- | --- | --- | --- | --- | --- | --- | --- | --- | --- | --- | --- |
| **Malawi** (n= 60) | Karonga (n =13) | 3 | 0 | 0.69 | 0.02 | 1 | 11 | 0.39 | 0.89 | -0.01 | 0.47 | 3 | 0.31 | 0.64 | 0.09 | -0.3 | 6 | 0.1 | 0.83 | 0.01 | 0.9 |
|  | Majete (n = 26) | 3 | 0 | 0.51 | 0.15 | 0.73 | 12 | 0.27 | 0.86 | -0 | 0.52 | 3 | 0.08 | 0.65 | -0 | 0.1 | 12 | 0.2 | 0.89 | 0.02 | 0.5 |
|  | Likoma (n = 21) | 3 | 0 | 0.64 | 0.14 | 0.62 | 11 | 0.43 | 0.88 | -0.03 | 0.69 | 5 | 0.43 | 0.75 | 0.06 | **0.7** | 11 | 0.3 | 0.9 | 0 | 0.7 |
| **Mozambique** (n = 70) | Maciana (n = 34) | 3 | 0.2 | 0.65 | -0 | 0.56 | 7 | 0.71 | 0.78 | -0.02 | 1 | 7 | 0.41 | 0.83 | 0.03 | 0.4 | 4 | 0 | 0.48 | -0.01 | -0 |
|  | Matola (n = 36) | 4 | 0.1 | 0.63 | 0.3 | 0.85 | 7 | 0.78 | 0.75 | -0.03 | 1 | 5 | 0.44 | 0.67 | 0.18 | -0.1 | 3 | 0 | 0.21 | -0.01 | -0 |
| **Uganda** (n = 63) | Agule (n = 13) | 3 | 0.3 | 0.57 | 0.35 | 0.25 | 8 | 0.39 | 0.85 | -0.05 | 0.85 | 10 | 0.85 | 0.88 | -0 | -0 | 10 | 0.3 | 0.92 | 0.01 | 0.1 |
|  | Lira (n = 12) | 3 | 0.3 | 0.65 | 0.33 | -0 | 7 | 0.1 | 0.86 | -0.05 | 0.51 | 8 | 0.7 | 0.87 | 0.07 | -0.1 | 9 | 0.7 | 0.91 | -0 | 0.3 |
|  | Apac (n = 18) | 3 | 0.1 | 0.46 | 0.01 | -0 | 7 | 0.05 | 0.8 | 0.06 | 0.91 | 13 | 0.75 | 0.89 | 0.02 | -0 | 13 | 0.5 | 0.9 | 0.02 | 0.7 |
|  | Kamuli (n = 20) | 5 | 0.5 | 0.79 | 0.23 | 0.04 | 11 | 0.11 | 0.88 | 0.03 | 0.86 | 7 | 0.33 | 0.66 | -0 | 0.5 | 12 | 0.4 | 0.91 | -0.01 | 0.4 |
| **Zambia** (n =60) | Nchelenge (n =30) | 3 | 0.3 | 0.6 | 0.02 | -0.2 | 10 | 0.27 | 0.84 | 0.01 | 0.85 | 7 | 0.5 | 0.83 | 0.01 | 0.2 | 9 | 0.5 | 0.83 | 0.01 | 0.2 |
|  | Namwala (n = 30) | 3 | 0.2 | 0.41 | 0.02 | -0.2 | 6 | 0.07 | 0.74 | 0.06 | 0.8 | 6 | 0.37 | 0.78 | -0 | 0.3 | 9 | 0.4 | 0.85 | -0.01 | 0.3 |
| **Zimbabwe** (n=70) | Mangwanda (n= 35) | 9 | 0.2 | 0.83 | 0.18 | 0.59 | 7 | 0.24 | 0.84 | 0.01 | 0.6 | 4 | 0.33 | 0.69 | -0 | 0.1 | - | - | - | - | - |
|  | Honde (n = 35) | 6 | 0.1 | 0.81 | -0 | 0.83 | 7 | 0.22 | 0.8 | 0.01 | 0.63 | 10 | 0.41 | 0.82 | 0.06 | 0.3 | - | - | - | - | - |

**Table S1 cont’d:** Population comparisons, HW proportions and independence of loci (significant values recorded in bold)

| **Country** | **Site / Locus** | **Overall Mean Na** | **Overall Mean Ho** | **Overall Mean H_E_** | **Overall Mean F_ST_** | **Overall Mean F_IS_** |
| --- | --- | --- | --- | --- | --- | --- |
| Malawi (n= 60) | Karonga (n =13) | 5.7 | 0.378 | 0.76 | 0.036 | 0.418 |
|  | Majete (n = 26) | 7.3 | 0.263 | 0.766 | 0.037 | 0.331 |
|  | Likoma (n = 21) | 7.2 | 0.333 | 0.795 | 0.039 | 0.34 |
| Mozambique (n = 70) | Maciana (n = 34) | 6 | 0.417 | 0.707 | 0.045 | 0.243 |
|  | Matola (n = 36) | 5.3 | 0.412 | 0.647 | 0.117 | 0.319 |
| Uganda (n = 63) | Agule (n = 13) | 6.3 | 0.314 | 0.759 | 0.066 | 0.394 |
|  | Lira (n = 12) | 5.8 | 0.367 | 0.777 | 0.046 | 0.409 |
|  | Apac (n = 18) | 7.5 | 0.304 | 0.75 | 0.088 | 0.502 |
|  | Kamuli (n = 20) | 7.5 | 0.329 | 0.785 | 0.013 | 0.492 |
| Zambia (n =60) | Nchelenge (n =30) | 9.3 | 0.373 | 0.789 | -0.009 | 0.388 |
|  | Namwala (n = 30) | 7.9 | 0.309 | 0.752 | 0.026 | 0.442 |
| Zimbabwe (n=70) | Mangwanda (n= 35) | 7.5 | 0.311 | 0.755 | 0.034 | 0.348 |
|  | Honde (n = 35) | 7 | 0.231 | 0.74 | 0.043 | 0.503 |
