# Supplementary figures and images for: Genetic differentiation and population structure of Anopheles funestus from Uganda and the southern African countries of Malawi, Mozambique, Zambia and Zimbabwe
Source: Parasit Vectors. 2020 Feb 18;13:87. doi: 10.1186/s13071-020-3962-1 (PMC7029513; doi:10.1186/s13071-020-3962-1)

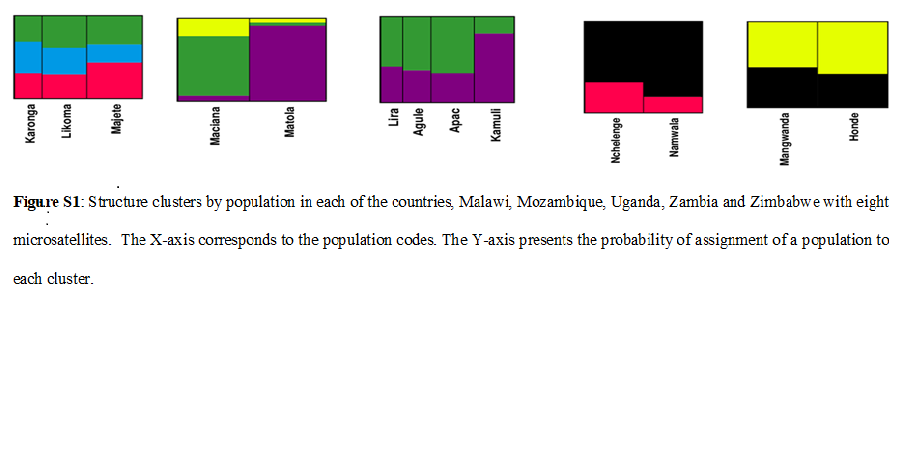

Supplement: Supplementary file 2 — Additional file 2: Figure S1. Structure clusters by population in each of the countries, Malawi, Mozambique, Uganda, Zambia and Zimbabwe with eight microsatellites. The X-axis corresponds to the population codes. The Y-axis presents the probability of assignment of a population to each cluster. [file 13071_2020_3962_MOESM2_ESM.tif]
